# The G-308A polymorphism of the TNF-α gene does not predict changes in cardiac function in response to medical therapy for idiopathic dilated cardiomyopathy

**Published:** 2008-10

**Authors:** Richard Brooksbank, Danelle Badenhorst, Gavin Norton, Angela Woodiwiss, Karen Sliwa

**Affiliations:** Cardiovascular Pathophysiology and Genomics Research Unit, School of Physiology, University of the Witwatersrand and Chris Hani-Baragwanath Hospital, Johannesburg; Cardiovascular Pathophysiology and Genomics Research Unit, School of Physiology, University of the Witwatersrand and Chris Hani-Baragwanath Hospital, Johannesburg; Cardiovascular Pathophysiology and Genomics Research Unit, School of Physiology, University of the Witwatersrand and Chris Hani-Baragwanath Hospital, Johannesburg; Cardiovascular Pathophysiology and Genomics Research Unit, School of Physiology, University of the Witwatersrand and Chris Hani-Baragwanath Hospital, Johannesburg; Department of Cardiology, University of the Witwatersrand and Chris Hani-Baragwanath Hospital, Johannesburg

## Abstract

**Summary:**

The G-308A polymorphism of the tumour necrosis factor-α (TNF-α) gene, a variant that influences TNF-α transcription, may contribute to non-ischaemic dilated cardiomyopathy. To evaluate whether TNF-α genotyping may assist in identifying a subset of patients who could potentially benefit from immunomodulatory therapy, we assessed the relationship between the G-308A polymorphism of the TNF-α gene and changes in left ventricular (LV) chamber dimensions and systolic function in patients with idiopathic dilated cardiomyopathy (IDC) before and six months after diuretic, digoxin and angiotensin-converting enzyme inhibitor (ACEI) therapy.

In 331 patients with IDC and 349 controls, the TNF-2 (A) allele (odds ratio = 1.509, 95% CI = 1.130–2.015, *p* < 0.01) and the TNF-12/22 (AG/GG) genotype (odds ratio = 1.620, 95% CI = 1.159–2.266, *p* < 0.01) were associated with IDC. However, in 122 patients with IDC, the TNF-α genotype was not associated with plasma TNF-α concentrations. In 133 patients with IDC, the TNF-α genotype failed to predict either the severity of pump dysfunction and cardiac dilatation at baseline, or changes in pump function and cardiac dimensions after six months of medical treatment.

We conclude therefore that although the TNF-α gene G-308A polymorphism may contribute to the development of IDC, it does not influence pump function or adverse cardiac remodelling in patients with IDC. Genotyping for this variant is therefore unlikely to assist in identifying patients with heart failure who may be particularly susceptible to novel immunomodulatory therapeutic strategies.

## Summary

Patients with heart failure have elevated plasma concentrations of the pro-inflammatory cytokine TNF-α.^1^ TNF-α may mediate a variety of detrimental effects on the heart, including cardiac dilatation, a decreased cardiac contractility^2^ and alterations in the properties of the extracellular matrix.^3^ Despite evidence in favour of a pathophysiological role of TNF-α in heart failure, therapeutic strategies designed to target TNF-α effects in heart failure have produced conflicting outcomes.^4^-^6^

Prior to further studies being performed with immunomodulatory therapy, it is therefore important to identify patients who are most likely to benefit from a decrease in TNF-α concentrations. In this regard, the genetic background may promote the deleterious effects of TNF-α in heart failure. Indeed, the TNF-2 (-308A) allele of a G-308A polymorphism of the TNF-α gene has been shown to be associated with the presence of non-ischaemic dilated cardiomyopathy in some studies.^7^,^8^

The TNF-2 allele is a more powerful transcriptional activator than the TNF-1 (G-308) allele and results in a seven-fold increase in induced TNF-α gene transcription.^9^ The TNF-2 allele also increases the sensitivity of white blood cells to endotoxin-stimulated white cell TNF-α production,^10^ which may be a significant source of TNF-α in more severe heart failure.^11^ It is therefore possible that patients with non-ischaemic dilated cardiomyopathy who have the TNF-2 allele may be a group who are particularly at risk for worsening heart failure and hence could be more susceptible to the potential benefits of immunomodulatory therapy. Although there are data to suggest that the G-308A polymorphism of the TNF-α gene is not associated with TNF-α concentrations in heart failure,^7^,^12^ the impact of the gene variant could still occur at a tissue level and subsequently modify cardiac effects.

There are no prospective data to determine the impact of this polymorphism on outcomes or disease progression in heart failure. In the present study, we therefore explored the possibility that the TNF-2 allele of the G-308A polymorphism of the TNF-α gene is associated with changes in left ventricular (LV) dimensions and pump function after six months of medical therapy in patients with idiopathic dilated cardiomyopathy (IDC).

## Methods

The Committee for Research on Human Subjects of the University of the Witwatersrand approved the study (approval number: M951122). Subjects provided informed consent.

The clinical component of the study was conducted between 1995 and 2001 when the use of β-AR blockers was not considered standard therapy for heart failure in South Africa. Three hundred and thirty-one patients with IDC and 349 age- and gender-matched control subjects of the same ethnic origin (African ancestry) were recruited. Patients were recruited if they were between 18 and 70 years of age, in NYHA functional class (FC) II or III heart failure of unknown aetiology, had a left ventricular ejection fraction (LVEF) < 40% as determined by radionuclide ventriculography, and had high-quality echocardiographic images with an LV end-diastolic diameter (LVEDD) > 5.5 cm. Exclusion criteria included evidence of another cause of heart failure and the presence of arrhythmias that could alter LVEF.

After initial presentation, and following a diagnosis by clinical examination and echocardiography (screening visit), 176 of the 331 patients agreed to participate in a prospective study assessing the impact of the TNF-α gene polymorphism on LV dimensions and function. During the six-month period of follow up, 24 patients died and 20 were lost to follow up. Of the remaining 132 patients who were followed prospectively, 71 were newly diagnosed.

All patients received treatment with digoxin and diuretics (furosemide) for seven days and then angiotensin-converting enzyme inhibitors (ACEI) were added to their therapy. The patients were followed for six months. Monthly visits were scheduled for clinical assessment and evaluation of the patients’ adherence to therapeutic agents. Clinical examinations, echocardiographic assessments and radionuclide studies were performed at baseline, and then repeated at six months.

The primary endpoints were LVEF, determined using radionuclide ventriculography, and LV end-diastolic diameter (LVEDD), determined using echocardiography. Radionuclide ventriculography as opposed to echocardiography was used as the method of preference to assess the impact of TNF-α genotype on LV systolic function, as this measurement is not subject to observer bias. To show a 10-point difference in radionuclide LVEF between groups with 80% power after six months of therapy required a sample size of 21 patients in each group.

## Functional class, echocardiography and radionuclide studies

A physician assessed the NYHA functional class of the patients during the baseline and follow-up visits. The same physician evaluated all patients. A multiple-gated equilibrium cardiac blood pool scintigraphic technique was used to measure LVEF (Elscint Apex 409).^13^ Imaging was performed in the left anterior oblique projection, providing the best septal separation of the ventricles with a 0–10° caudal angulation. Calculations of LV performance were made as previously described,^14^ using an automatic edge-detection algorithm for the determination of LV borders. A single observer interpreted all studies.

Two-dimensional targeted M-mode echocardiography with Doppler colour flow mapping was performed using a Hewlett Packard Sonos 5500 echocardiograph attached to a 2.5- or 3.5-MHz transducer. All studies were performed and interpreted by the same operator and recorded on videotape. Left ventricular dimensions were measured according to the American Society of Echocardiography guidelines.^15^ Measurements of LV dimensions and function were determined over an average of three or more beats. The investigators that performed and interpreted the radionuclide and echocardiographic studies were unaware of the genotype of the patients.

## Genotyping

Genomic DNA was extracted from white blood cells as described by Higuchi *et al.*^16^ The following PCR primers were used to amplify a 107-bp fragment containing the G-308A TNF-α polymorphism: forward primer: 5′-AGGCAATAGGTTTTGAGGGCCAT-3′ reverse primer: 5′-TCCTCCCTGCTCCGATTCCG-3′. Reaction conditions were one cycle of 94°C for three minutes, followed by 35 cycles of 94°C for 30 seconds, 60ºC for 30 seconds and 72ºC for one minute. Each PCR reaction contained 200–500 ng of genomic DNA, 1U Taq DNA polymerase (Takara), 1.5 mM MgCl_2_, 200 nM of each primer, 200 μM of each dNTP and 50 mM KCl. The G-308 substitution gives rise to a *Nco* I restriction enzyme recognition site and therefore, following PCR amplification, the 107 bp PCR product was digested with 5U of *Nco* I (Roche) for four hours at 37ºC and the resulting DNA fragments were visualised on a 3.5% agarose gel. The G allele gives rise to two digestion products of 87 and 20 bp, therefore TNF-11 homozygotes show two bands of 87 and 20 bp, TNF-12 heterozygotes show three bands of 107, 87 and 20 bp and TNF-22 homozygotes show a single band of 107 bp.

## Determination of plasma TNF-α concentration

Plasma TNF-α concentration was determined in the first consecutive 122 patients using a high-sensitivity, human TNF-α-specific ELISA kit (Amersham) according to the manufacturer’s instructions.

## Data analysis

Data are presented as mean ± SEM. Case and control group mean values were compared with the use of a two-sample Student’s *t*-test or a Mann-Whitney test, depending on whether variables were nominal or ordinal (Bartlett’s test). To test for Hardy-Weinberg equilibrium, the expected genotype numbers were calculated from the allele frequencies and deviation from the observed genotype numbers was determined using a χ^2^ test. The relative risk of the presence of IDC with the TNF-α G-308A polymorphism was estimated from an odds ratio calculation using a Fisher’s exact test.

To assess the effect of genotype on either baseline LVEF or cardiac dimensions, a MANCOVA was performed with age, gender, disease duration and BMI included in the regression model. A paired Student’s *t*-test was used to detect changes from baseline. Analysis of covariance adjusting for baseline data, age, gender, disease duration and type of ACEI was employed to determine differences in changes in LV cavity size and function, and final LV cavity size and function between genotype groups. Genotype effects on final LV cavity size and function were assessed using MANCOVA with age, gender, BMI, disease duration and baseline data included as covariates. Data are expressed as mean ± standard error of the mean (SEM).

## Results

The characteristics of the patient and control groups are summarised in Table 1. There were no differences in the demography and clinical characteristics between the case and control groups, although the patient group did contain a slightly higher proportion of males. The subgroup of patients on whom follow-up LV structure and function were assessed was comparable in demography and clinical characteristics to that of the total group of patients assessed. The characteristics of genotype-specific subgroups in patients in whom LV structure and function were determined are summarised in Table 2. No differences were noted in the demographic or general clinical data.

**Table 1:** Characteristics Of Patients With Idiopathic Dilated Cardiomyopathy (IDC) And Control Groups

	*IDC*	*Control*
	*(n = 331)*	*(n = 349)*
Age (years)	51.2 ± 0.9	49.5 ± 0.5
Gender (% male)	62	56
Body mass index (kg.m^-2^)	25.1 ± 0.4	25.2 ± 0.3
Systolic blood pressure (mmHg)	119 ± 1	126 ± 1
Diastolic blood pressure (mmHg)	76 ± 1	78 ± 1
LV ejection fraction (%)	26.3 ± 0.7	–
LV end-diastolic diameter (mm)	65.7 ± 0.7	–
Plasma TNF-α (pg.ml^-1^)	7.66 ± 0.74*	–

*In 122 patients

**Table 2:** Baseline Demographic And Clinical Characteristics Of Patients With Idiopathic Dilated Cardiomyopathy (IDC), Grouped AccordingTo Tumour Necrosis Factor-α Genotype

	*Genotype group*	
	*11*	*12 + 22*
	*(n = 218)*	*(n = 113)*
Age (years)	50.8 ± 0.8	51.8 ± 1.1
Gender (% male)	62	57
Body mass index (kg.m^-2^)	25.1 ± 0.5	25.1 ± 0.7
Systolic BP (mmHg)	118 ± 1	118 ± 2
Diastolic BP (mmHg)	76 ± 1	77 ± 2
Functional class (I/II/III/IV) (%)	4/42/46/8	3/37/50/10
Disease duration (months)	11.0 ± 2.1	9.8 ± 2.3
Perindopril/enalapril/trandolapril (%)	20/35/45	25/42/33

The genotype and allele frequencies are shown in Table 3. When comparing expected and actual genotype numbers, the control group was estimated to be in Hardy-Weinberg equilibrium with a χ^2^ value of 5.11, whereas the patient group, with a χ^2^ value of 23.72, was not in Hardy-Weinberg equilibrium. A dominant model of inheritance of the risk allele was assumed, and the TNF-12 and TNF-22 genotypes groups were pooled. A significant excess of both the TNF-12 and 22 genotypes [odds ratio = 1.620 (95% CI = 1.159–2.266), *p* < 0.01] and the TNF-2 allele [odds ratio = 1.509 (95% CI = 1.130–2.015), *p* < 0.01] was noted in the IDC group compared to the control group, suggesting that the TNF-2 allele is associated with the development of IDC in the population studied.

**Table 3:** Genotype And Allele Frequencies Obtained For The TNF-A G-308A Polymorphism In Patients With Idiopathic Dilated Cardiomyopathy (IDC) And Controls

	*Genotype*			*Allele*	
	*11*	*12*	*22*	*1*	*2*
IDC (*n* = 331)	218 (0.66)	6 (0.29)	16 (0.05)	532 (0.81)	128 (0.19)
Control (*n* = 349)	265 (0.76)	72 (0.21)	12 (0.03)	602 (0.86)	96 (0.14)

Numbers in parentheses represent frequencies. Odds ratios and confidence intervals for relationships between genotype and IDC are given in the text.

At baseline, the G-308A TNF-α polymorphism was not associated with either LV dimensions or systolic function (Table 4). Moreover, no relationship between the TNF-2 genotypes and baseline plasma TNF-α concentrations was noted in patients with IDC (TNF-11 = 7.9 ± 0.9 pg.ml^-1^ vs TNF-12/22 = 7.2 ± 0.9 pg.ml^-1^).

**Table 4:** Left Ventricular (LV) Chamber Dimensions And Function In Patients With Idiopathic Dilated Cardiomyopathy (IDC) Prospectively Studied, Grouped According To Tumour Necrosis Factor-α Genotype

	*Genotype group*					
	*11 (n = 88)*			*12/22 (n = 46)*		
	*Baseline*	*Final*	*Mean change*	*Baseline*	*Final*	*Mean change*
LV end-diastolic diameter (mm)	66.8 ± 0. 8	63.4 ± 1.0	–2.4 ± 0.7	66.5 ± 1.2	62.7 ± 1.2	–3.2 ± 1.0
LV end-systolic diameter (mm)	58.1 ± 0.8	53.9 ± 1.2	–4.1 ± 1.0	58.4 ± 2.2	53.2 ± 1.6	–4.7 ± 1.3
LV ejection fraction (%)	22.8 ± 0.8	30.7 ± 1.3	7.4 ± 1.2	22.9 ± 1.2	27.9 ± 1.6	5.1 ± 1.4

A similar percentage of patients in each TNF-α genotype-specific group died or was lost to follow-up (data not shown). There was also a similar percentage of newly diagnosed patients in each group. Both genotype groups received similar doses and type of drug therapy (type of ACEI is indicated in Table 2). Following six months of therapy, LV ejection fraction (LVEF) (MUGA) increased by 6.6 ± 0.9 absolute units (*p* < 0.0001), LV end-diastolic diameter (LVEDD) decreased by 2.7 ± 0.6 mm (*p* < 0.02), and LV end-systolic diameter (LVESD) decreased by 4.3 ± 0.7 mm (*p* < 0.001) in all patients considered together. The increase in LVEF and decreases in LVEDD and LVESD were the same in TNF-α genotype-specific groups (Table 4). TNF-α genotype failed to predict final LV function and cavity dimensions (Table 4).

## Discussion

The main findings reported in the present study are that although the TNF-12/22 genotypes and the TNF-2 allele of the TNF-α G-308A polymorphism were associated with the presence of IDC, the TNF-2 allele did not predict on-treatment LV systolic function and internal dimensions over six months of therapy in patients with IDC.

It is well established that systemic inflammation and inflammatory mediators play an important role in the pathogenesis and progression of heart failure.^17^ The TNF-2 allele of the G-308A polymorphism, given that it is associated with increased inducible TNF-α production,^9^,^10^ could therefore contribute to the deleterious changes noted to occur in heart failure by enhancing TNF-α concentrations at either a circulating or a tissue level. In the present study we were unable to show a relationship between the TNF-12/22 genotype and plasma TNF-α concentrations, a finding that nevertheless does not preclude an effect on tissue TNF-α expression. More importantly however, we were unable to show a relationship between the TNF-2 allele and on-treatment LV dimensions and systolic function, a finding that suggests that even if the TNF-α genotype influences TNF-α tissue expression, this fails to translate into significant clinical changes.

The present study is the first prospective study to explore the possibility that the TNF-2 allele contributes to adverse cardiac remodelling and cardiac dysfunction in heart failure. Although a prior study has suggested that the TNF-2 allele does not influence cardiac function or dimensions in heart failure,^18^ this study was cross-sectional and not prospective in nature. The outcomes of that study^18^ could, therefore, have been considerably influenced by differences in current therapy. In contrast, in the present study, cardiac dimensions and function were assessed in patients on treatment with the same classes of agents over a six-month treatment period. The lack of association between the TNF-2 allele and cardiac function and dimensions in the present study therefore suggests that the TNF-2 allele is unlikely to assist in identifying patients with heart failure who may be particularly susceptible to novel immunomodulatory therapeutic strategies.

The lack of association between the TNF-α G-308A polymorphism and plasma TNF-α concentrations in the present study is consistent with a lack of association noted in prior studies in either IDC^7^ or heart failure in general.^12^ This finding suggests that in IDC, either the TNF-2 allele is suppressed or the effect of the TNF-2 allele is overridden by other factors associated with heart failure, such as disruption of the cytokine network.^19^

It may be argued that the small number of individuals who were found to be homozygous for the TNF-2 allele in the present study may have prevented us from identifying an impact of the TNF-2 allele acting in a recessive manner on cardiac dimensions, systolic function and plasma TNF-α concentrations. However, previous studies have demonstrated an increased white cell TNF-α production in heterozygotes for the G-308A polymorphism.^10^ Moreover, in heart failure attributed mainly to ischaemic causes, plasma TNF-α concentrations are elevated to a similar degree in TNF-2 homozygotes compared to TNF-12 heterozygotes and TNF-1 homozygotes.^12^

The finding in the present study that both the TNF-12/22 genotype and the TNF-2 allele were associated with the presence of IDC in the population studied is consistent with previous studies,^7^,^8^ but in contrast to another report.^18^ An explanation for these discrepancies is not immediately apparent, but may be attributed to differences in populations studied or because of population stratification between cases and controls. Although modest sample sizes (~ 48–175 patients) may be criticised in the two previous studies showing an association between genotype and non-ischaemic cardiomyopathies,^7^,^8^ we studied a considerably greater study sample size (*n* = 331 cases and 349 controls).

Assuming that the TNF-2 allele is a risk factor for IDC, a potential explanation for the association could be that in IDC, a trigger event such as a viral infection could lead to excess cardiac TNF-α production in individuals carrying the TNF-2 allele, which in turn could augment cardiac remodelling and pump dysfunction. Viral infection is currently considered to be a likely trigger for the development of many cases of IDC.^20^

Although the present study was a prospective study where therapy was largely standardised between patients, there are still potential limitations to the study. In particular, prospective follow-up was for only six months, rather than a longer period. However, this period was selected because beyond six months, mortality and morbidity related to heart failure would have limited our ability to appropriately assess LV structural and functional changes.^21^

## Conclusion

The present study shows that although the TNF-2 allele may contribute to the development of IDC, this allele has no impact on in-treatment adverse chamber remodelling and pump dysfunction. Genotyping for this variant is therefore unlikely to assist in identifying patients with heart failure who may be particularly susceptible to novel immunomodulatory therapeutic strategies.

## References

[1] Levine B, Kalman J, Mayer L, Fillit HM, Packer M (1990). Elevated circulating levels of tumor necrosis factor in severe chronic heart failure.. N Engl J Med.

[2] Bozkurt B, Kribbs SB, Clubb FJ, Michael LH, Didenko VV, Hornsby PJ (1998). Pathophysiologically relevant concentrations of tumor necrosis factor-alpha promote progressive left ventricular dysfunction and remodeling in rats.. Circulation.

[3] Li YY, Feng YQ, Kadokami T, McTiernan CF, Draviam R, Watkins SC (2000). Myocardial extracellular matrix remodeling in transgenic mice overexpressing tumor necrosis factor alpha can be modulated by antitumor necrosis factor alpha therapy.. Proc Natl Acad Sci (USA).

[4] Chung ES, Packer M, Lo KH, Fasanmade AA, Willerson JT (2003). Anti-TNF Therapy Against Congestive Heart Failure investigators. Randomized, double-blind, placebo-controlled, pilot trial of infliximab, a chimeric monoclonal antibody to tumor necrosis factor-alpha, in patients with moderate-to-severe heart failure: results of the anti-TNF Therapy Against Congestive Heart Failure (ATTACH) trial.. Circulation.

[5] Anker SD, Coats AJ (2002). How to RECOVER from RENAISSANCE? The significance of the results of RECOVER, RENAISSANCE, RENEWAL and ATTACH.. Int J Cardiol.

[6] Sliwa K, Skudicky D, Candy G, Wisenbaugh T, Sareli P (1998). Randomised investigation of effects of pentoxifylline on left-ventricular performance in idiopathic dilated cardiomyopathy.. Lancet.

[7] Ito M, Takahashi H, Fuse K, Hirono S, Washizuka T, Kato K (2000). Polymorphisms of tumor necrosis factor-alpha and interleukin-10 genes in Japanese patients with idiopathic dilated cardiomyopathy.. Jpn Heart J.

[8] Densem CG, Hutchinson IV, Yonan N, Brooks NH (2002). Tumour necrosis factor alpha gene polymorphism: a predisposing factor to non-ischaemic myocardial dysfunction?. Heart.

[9] Wilson AG, Symons JA, McDowell TL, McDevitt HO, Duff GW (1997). Effects of a polymorphism in the human tumor necrosis factor alpha promoter on transcriptional activation.. Proc Natl Acad Sci (USA).

[10] Louis E, Franchimont D, Piron A, Gevaert Y, Schaaf-Lafontaine N, Roland S (1998). Tumour necrosis factor (TNF) gene polymorphism influences TNF-alpha production in lipopolysaccharide (LPS)-stimulated whole blood cell culture in healthy humans.. Clin Exp Immunol.

[11] Niebauer J, Volk HD, Kemp M, Dominguez M, Schumann RR, Rauchhaus M (1999). Endotoxin and immune activation in chronic heart failure: a prospective cohort study.. Lancet.

[12] Kubota T, McNamara DM, Wang JJ, Trost M, McTiernan CF, Mann DL, Feldman AM (1998). Effects of tumor necrosis factor gene polymorphisms on patients with congestive heart failure. VEST Investigators for TNF Genotype Analysis. Vesnarinone Survival Trial.. Circulation.

[13] Pitt B, Strauss HW (1977). Evaluation of ventricular function by radioisotopic techniques.. N Engl J Med.

[14] Reiber JHC (1985). Quantitative analysis of left ventricular function from equilibrium gated blood scintigrams: an overview of computer methods.. Eur J Nucl Med.

[15] Sahn DJ, de Maria A, Kisslo J, Weyman A (1978). The committee on Mmode standardization of the American Society of Echocardiography: Recommendations regarding quantitation in M-mode echocardiography: results of a survey of echocardiographic measurements.. Circulation.

[16] Higuchi R (1989). Rapid and efficient DNA extraction for PCR from cells or blood.. Amplifications.

[17] Yndestad A, Damås JK, Oie E, Ueland T, Gullestad L, Aukrust P (2006). Systemic inflammation in heart failure--the whys and wherefores.. Heart Fail Rev.

[18] Tiret L, Mallet C, Poirier O, Nicaud V, Millaire A, Bouhour JB (2000). Lack of association between polymorphisms of eight candidate genes and idiopathic dilated cardiomyopathy: the CARDIGENE study.. J Am Coll Cardiol.

[19] Aukrust P, Ueland T, Lien E, Bendtzen K, Muller F, Andreassen AK (1999). Cytokine network in congestive heart failure secondary to ischemic or idiopathic dilated cardiomyopathy.. Am J Cardiol.

[20] Fujioka S, Kitaura Y, Deguchi H, Shimizu A, Isomura T, Suma H, Sabbah HN (2000). Lack of association between polymorphisms of eight candidate genes and idiopathic dilated cardiomyopathy: the CARDIGENE study.. J Am Coll Cardiol.

[21] (1991). Effect of enalapril on survival in patients with reduced left ventricular ejection fractions and congestive heart failure.. New Engl J Med.

